# Dengue in Adults Admitted to a Referral Hospital in Hanoi, Vietnam

**DOI:** 10.4269/ajtmh.14-0472

**Published:** 2015-06-03

**Authors:** Walter R. Taylor, Annette Fox, Khuong Thi Pham, Hoa Nguyen Minh Le, Ninh Thi Hai Tran, Giang Van Tran, Binh Thanh Nguyen, My Van Nguyen, Lien Thi Nguyen, Sophie Yacoub, Hoai Thi Thu Nguyen, Ha Hong Nguyen, Hien Duc Nguyen, Heiman Wertheim, Peter Horby, Jeremy Farrar, Lien Thi Minh Trinh, Kinh Van Nguyen

**Affiliations:** Oxford University Clinical Research Unit (OUCRU), Wellcome Trust Major Overseas Programme, Hanoi, Socialist Republic of Vietnam; Nuffield Department of Clinical Medicine, Centre for Tropical Medicine, University of Oxford, Oxford, England; National Hospital for Tropical Diseases, Hanoi, Socialist Republic of Vietnam; Department of Medicine, Imperial College, Hammersmith Campus, London, England; Vietnam National Heart Institute, Bach Mai hospital, Hanoi, Socialist Republic of Vietnam; Oxford Clinical Research Unit, Hospital for Tropical Diseases, Ho Chi Minh City, Socialist Republic of Vietnam

## Abstract

Knowledge of adult dengue virus (DENV) infection from Hanoi, Vietnam, is limited. In 2008, we prospectively studied 143 (77 male) confirmed (nonstructural 1 antigen enzyme-linked immunosorbent assay [ELISA], DENV polymerase chain reaction, paired serology) adult dengue patients of median age 23.5 (range 16–72) years. They were admitted to the National Hospital for Tropical Diseases, Hanoi, on median illness day (D) 5 (range 1–8). By D8, 141 (98.6%) were afebrile. Platelet counts and hematocrit (median, interquartile range [IQR]) nadired and peaked on D5 and D4, respectively: 40,000/μL (10,000–109,000/μL), 43.4% (34.9–49.7%). Four (2.8%) patients had severe dengue: 1) D10 shock (*N* = 1) and 2) aspartate aminotransferase (AST) ≥ 1,000 IU/L (*N* = 3, D5 and D7). Of 143 patients, 118 (82.5%) had ≥ 1 warning sign (World Health Organization [WHO] 2009 criteria): mucosal bleeding 66/143 (46.1%), soft tissue edema 54/143 (37.7%), and ultrasound detected plasma leakage (pleural effusions/ascites) 30/129 (23.25%). 138 (96.5%) patients received intravenous (IV) fluids: 3 L (IQR: 0.5–8.5 L). Most patients had non-severe dengue with warning signs. High rates of edema and plasma leakage may be explained partly by liberal use of IV fluids. Studies are needed on optimizing fluid management in non-severe adult dengue.

## Introduction

Serotypes 1–4 of the dengue flavivirus cause dengue, a predominantly tropical infection transmitted to humans by the *Aedes* mosquito. Most dengue infections are asymptomatic,^1^,^2^ and symptomatic dengue is usually an acute, self-limiting undifferentiated, or exanthematous febrile illness. However, some patients may experience an increase in systemic vascular permeability that results in the intravascular loss of plasma (plasma leakage), which may manifest as soft tissue edema, serous effusions, hypotension, shock, and bleeding.^3^–^5^ Impaired systolic and diastolic cardiac function may also occur contemporaneously and contribute to cardiovascular instability.^6^ This critical phase occurs usually several days after patients are afebrile on illness days (D) 4–7 and lasts 24–48 hours.^7^

In 2009, updated World Health Organization (WHO) guidelines classified dengue as severe or non-severe to reflect its changing clinical epidemiology better.^8^–^12^ Severe dengue is characterized by any one of plasma leakage leading to shock or respiratory distress, clinically severe bleeding, aspartate aminotransferase (AST) ≥ 1,000 IU/L, impaired consciousness, and other organ involvement (e.g., heart, kidney). Shock is the most common manifestation of severe dengue, affecting 210/230 (91.3%) patients in the Dengue Control (DENCO) study,^12^ and is more common in children. The median age in the DENCO study was 11.5 years (Thomas Janish, personal communication), and in southern Vietnam, shock was about threefold more common in children compared with adults.^13^ Dengue warning signs (Box 1) in non-severe dengue may herald the development of severe dengue, thus forewarning clinicians to monitor their patients closely.^12^

Box 1

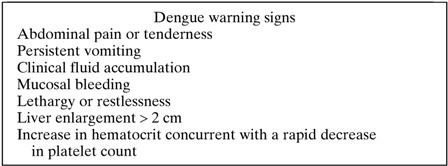

Common dengue-induced laboratory changes include thrombocytopenia, leukopenia, atypical lymphocytes, immature neutrophils, raised hematocrit (Hct) and liver enzymes, mild increases in prothrombin time (PT) and activated partial thromboplastin time (aPTT), and increased fibrinolysis.^14^,^15^ Alanine transaminase (ALT)/AST increase tend to be mild to moderate (2.5–5 × upper limit of normal [ULN]), but acute liver failure with severe bleeding may occur.^5^,^12^,^16^,^17^

In Vietnam, all four dengue serotypes are transmitted.^13^,^18^ Serotypes 1 and 2 predominate,^19^–^21^ but serotype 4 has emerged recently as the main serotype in southern Vietnam.^18^ Dengue transmission in northern Vietnam is less intense than the tropical southern regions, but dengue notifications have increased annually since 1999.^22^ It is common in young adults with median age 23 years, as found in one study from Hanoi.^22^ This is epidemiologically similar to other southeast (SE) Asian countries and countries in Latin America.^23^,^24^

Given the importance of adult dengue in Hanoi and the lack of data describing its clinical and laboratory features, we conducted the observational study reported herein, classifying patients according to the 2009 WHO criteria.^8^

## Materials and Methods

### Study site and patient population.

The study took place from September to November 2008 at the National Hospital for Tropical Diseases (NHTD), in Hanoi, an adult tertiary referral hospital. Admission numbers for clinically suspected dengue have varied from < 100/year to up to 5,000 in 2009 (NHTD data). In an earlier analysis, our patients had DENV serotypes 1 (32%) and 2 (30%), and serological findings were consistent with primary and secondary infections in 34% and 61% patients, respectively.^19^

Patients were enrolled into the study if they gave signed, informed consent and had clinically suspected dengue,^25^ that is, a history of fever and more than two of the following symptoms: 1) headache, 2) retro-orbital pain, 3) myalgia, 4) arthralgia, 5) rash, 6) hemorrhagic manifestations, and 7) leukopenia. Ethical approvals were obtained from the Scientific and Ethical Committee of the NHTD and the Oxford University Tropical Research Ethics Committee.

### Study conduct and clinical care.

Patients were managed by their attending physicians in the general infection ward. Enrolled patients underwent 1) history, 2) daily physical examinations, 3) 8 hourly pulse, blood pressure (BP), and axillary temperature (fluid input–output measurements were not done routinely), 4) a tourniquet test in patients without bleeding signs (e.g., cutaneous petechiae, mucosal bleeding), 5) daily full blood count, 6) routine biochemistry (admission, discharge, and as clinically indicated), 7) aPTT (normal range [NR] 26.3–39.4 seconds), 8) PT (NR: 70–140%), 9) fibrinogen (NR: 1.75–4 g/L), 10) dengue viral load and serotype by polymerase chain reaction (PCR), as previously reported,^26^,^27^ 11) dengue nonstructural (NS) 1 antigen (Platelia™ Dengue NS1 Ag, BIO-RAD, Hercules, CA, following the manufacturer's instructions), 12) dengue serology by IgM and IgG capture ELISA against DENV,^28^ and 13) abdominal and chest ultrasounds. Transthoracic echocardiograms with Doppler were performed at the Vietnam National Heart Institute close by on campus on a subset of patients.

Treatment included encouraging oral intake, intravenous (IV) fluids and platelet transfusions. National guidelines suggest platelet transfusions if either the platelet count is < 50,000/μL with significant bleeding or the platelet count is < 5,000/μL. Patients were discharged and asked to return for follow-up (generally after 1–2 weeks) for convalescent clinical and laboratory assessments.

### Definitions.

#### Dengue serology.

Serological samples were positive for IgM or IgG if the optical density (OD) units were ≥ 6 times higher than the negative control sera (i.e., units ≥ 12); all other results were classed as negative including “indeterminate” ratios of 8–< 12.^19^ A change from a negative to a positive result defined a seroconversion.

#### Confirmed dengue infection.

Confirmed DENV^8^ was any one of the following: 1) positive DENV PCR; 2) positive dengue NS1 antigen; and 3) paired serology that showed any one of the following: a) an IgM seroconversion, b) a rising IgM titer of ≥ 20%, c) an IgG seroconversion, and d) a ≥ fourfold rise in IgG titers. In addition, we considered a declining IgM titer when the convalescent sample had been taken on illness day (D) ≥ 14 as evidence of acute dengue infection; D14 is the mean time to peak IgM titers.^29^

#### Highly suggestive/probable dengue infection.

Highly suggestive/probable dengue infection was diagnosed if consistent clinical features were present and if paired serological tests showed one of the following: 1) static IgM titers, 2) an increase of IgM titers < 20%, 3) declining IgM titers when the convalescent sample was taken before illness D14, and 4) paired IgG titers showing a < 4 fold rise in titer. A single IgM positive serological sample was considered unclassifiable.

#### Severe dengue and dengue warning signs.

See Introduction and Box 1.

### Data management and statistics.

Data were recorded onto a case record form (CRF), double entered into Microsoft Access 2003 (Microsoft Corporation, Seattle, WA), and analyzed with Access and Stata v9 (STATA Corporation, College Station, TX). Trends in clinical and laboratory data are shown by illness day; D1 is the first day of reported fever. Proportional data were analyzed by two-sided χ^2^ or Fisher's exact test, continuous data by student's *t* test (normally distributed continuous data) or Mann–Whitney *U* (skewed data) test, as appropriate.

## Results

### Diagnosis and admission clinical characteristics.

**Figure 1.**
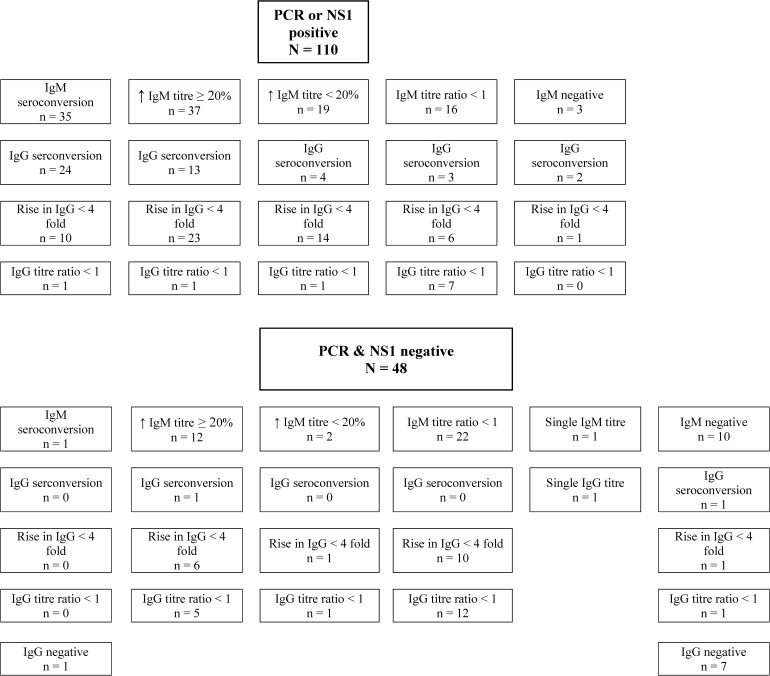
Serological responses in patients stratified by nonstructural 1 (NS1) antigen and polymerase chain reaction (PCR) positivity.

Of the 206 patients screened, 158 were enrolled: 1) 143 were dengue confirmed, 2) 5 had highly suggestive/probable dengue, and 3) 10 did not have dengue. The NS1 antigen test was positive in 96/158 (60.7%) patients and DENV PCR was positive in 81/158 (51.3%), together accounting for 110/143 (76.9%) confirmed dengue diagnoses. The IgM and IgG antibody responses varied and were similar between the NS1 and PCR positive and negative groups (Figure 1). In the latter, 14 patients had confirmed dengue by WHO serological criteria and 19 by our modified criterion. Analyses presented henceforth are for the 143 confirmed dengue patients.

The majority of patients came from Hanoi and their median age was 23.5 years; just over half were males. Patients were admitted to hospital on illness day D1–8 (median 5) and remained in hospital for a median of 5 (interquartile range [IQR] 2–12, range 0–14) days; 13 (9.1%) patients were hospitalized for > 1 week.

Prior to admission, 107/118 (90.7%) patients had received a variety of drugs, usually paracetamol, vitamins, and antibiotics and 70/115 (60.9%) had received IV fluids; 32/127 (25.2%) had been transferred from another hospital. Admission symptoms and signs are detailed in Table 1. Ascites and pleural effusions were detected by ultrasound in 4 (3.1%) of 129 patients and were not associated with a history of receiving preadmission IV fluids (*P* = 1).

### Trends in vital signs by day of illness.

The number of patients in the study by illness day is shown in Table 2. The median temperature fell over time and by D8 141/143 (98.6%) patients were afebrile. The median systolic, diastolic, and pulse pressures were similar across illness days and the median changes within patients (lowest recorded BP versus enrollment) were small −2.5 (IQR: −30 to 0) and 0 (IQR: −10 to 20) mmHg for the systolic and diastolic BPs, respectively. Pulse pressures of 20 mmHg were recorded in five patients; their clinical features are shown in Supplemental Table 1.

### Trends in hematology and biochemistry by day of illness.

**Figure 2.**
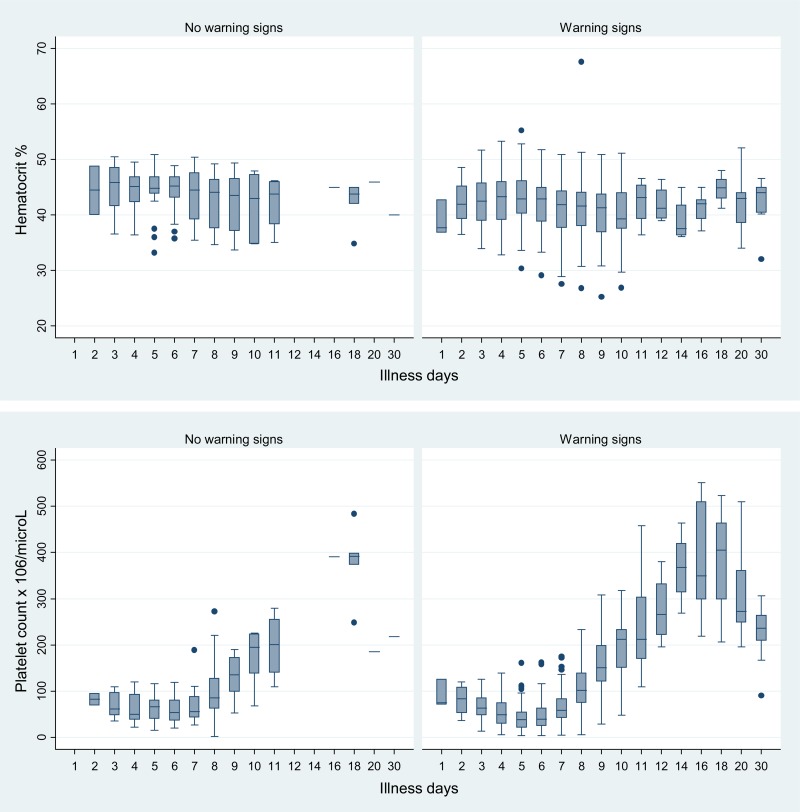
Trends in hematocrits and platelet counts by illness day as a function of the presence of warning signs. For clarity some illness days have been combined, and D30 includes laboratory values after day 30.

**Figure 3.**
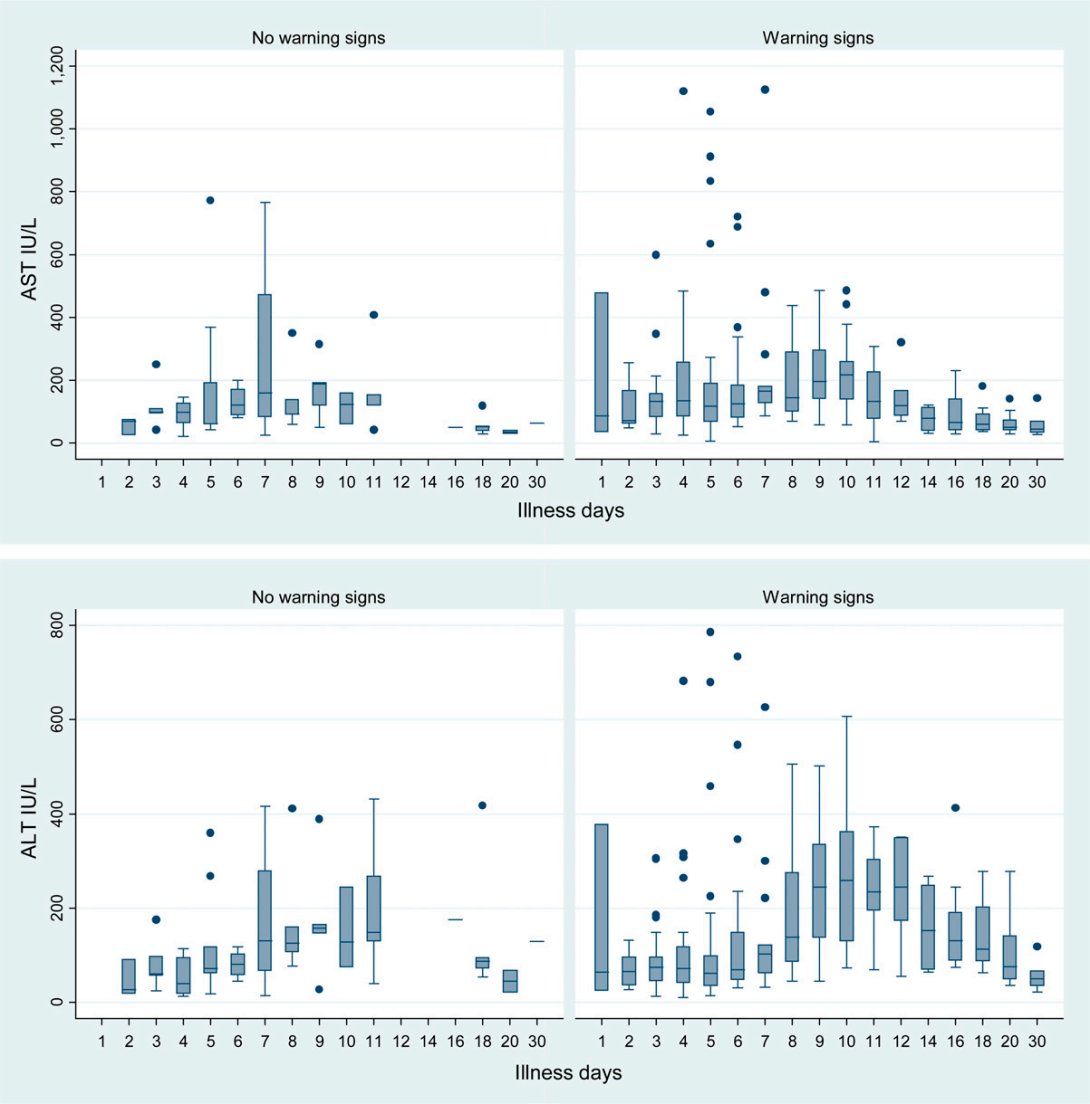
Trends in the aspartate aminotransferase (AST) and alanine transaminase (ALT) concentrations by illness day as a function of the presence of warning signs. For clarity some illness days have been combined, and D30 includes laboratory values after day 30.

Nadir or peak median values of hematologic and biochemical parameters occurred generally between D4 and D6 (Figures 2 and 3, Table 2). The median nadir platelet occurred on illness D5: 40,000/μL (IQR: 10,000–109,000/μL); for individual patients, the nadir platelet counts occurred on D6 (IQR: 5–6). D5 median platelet counts were significantly lower in those with 1) warning signs (*N* = 90) versus no warning signs (*N* = 17): 38,000 versus 67,000/μL (*P* = 0.0046) and 2) mucosal bleeding (*N* = 49) versus no mucosal bleeding (*N* = 58): 37,000 versus 47,500/μL (*P* = 0.037).

The highest median Hcts were recorded on D4, 43.4% (IQR: 34.9–49.7%), D5, 43.3% (IQR: 35–50.9%), and D6, 43.1% (IQR: 33.8–49.8%); in individual patients, peak Hcts were on D5 (IQR: 4–7). Most aPTTs and PTs were within normal limits but median times trended upward, peaking on D5 and D7, respectively (Table 2). Median fibrinogen concentrations fluctuated but were mostly below the lower limit of normal (LLN). There were no significant differences between the median values of these clotting parameters and the presence of warning signs or mucosal bleeding across all study days combined and on D4–D7 (data not shown).

The median total white cell count showed leukopenia (< 4,000/μL) for the first 5 days. Absolute lymphopenia (< 1,000/μL) was detected at least once in 76/143 (53.1%) patients, most frequently during the first 5 illness days (Table 1). Absolute neutropenia (< 1,500/μL) was detected at least once in 125/143 (87.4%) patients and persisted up to D11. Atypical lymphocytes and immature neutrophils were detected in 88 (61.5%) and 50 (34.9%) patients, respectively, most frequently on D10.

AST and ALT values varied widely (Figure 3). The highest ALT value was 786 IU/L and 8 patients had values ≥ 500 IU/L, including 3 patients with AST values > 1,000 IU/L. Of these eight patients, five had normal total bilirubin concentrations, two had missing data, and one (patient with AST 1,126 IU/L) had an elevated concentration of 21.7 mg/dL.

### Dengue warning signs.

Of the 143 patients, 122 (4 severe, 118 non-severe [85.3%]) had at least one dengue warning sign: abdominal pain or tenderness (*N* = 42/143 [29.4%]); persistent vomiting (*N* = 6, 4.2%); edema of face, hands, or feet (*N* = 54, 37.8%); mucosal bleeding (*N* = 66 (46.2%): nose/mouth (*N* = 35), subconjunctival (*N* = 35), vagina (*N* = 16); lethargy (*N* = 4, 2.8%); and a palpable liver (*N* = 10, 7%, liver size not measured). Ultrasounds, performed in 129 patients, detected 30 (23.3%) with ascites alone (*N* = 9), a pleural effusion alone (*N* = 14), or both (*N* = 7) from D4 to D14, median 7 (IQR: 5–11). The risk of developing ultrasound detected plasma leakage was 3.5-fold (95% CI: 1.3–9.6, *P* = 0.005) higher in those who received preadmission IV fluids (22/70 [31.4%]) versus those who had not (4/45 [8.9%]), but the median IV fluid volumes administered during admission were similar (*P* = 0.31) in patients with and without plasma leakage: 3 and 3.2 L, respectively. Patients with concurrent rising Hcts and declining platelet counts numbered 17 (11.9%).

### WHO defined severe dengue.

Four patients (2.8% [95% CI: 0.77–7.01]) developed severe dengue: 1) one [0.69 (0–3.8)%] developed shock (systolic BP 70 mmHg) on D10 and 2) three had AST > 1,000 IU/L but no clinical features of acute liver failure. All had prior warning signs. The shocked patient had facial swelling, abdominal pain, and ascites; the other three had mucosal bleeding (*N* = 1), lethargy (*N* = 1), and a pleural effusion (*N* = 1). No patients had severe bleeding, reduced consciousness, or clinical evidence of other end-organ damage, and none died.

### Echocardiograms.

Echocardiograms (*N* = 44) showed significantly higher median admission versus convalescent values for 1) right ventricular myocardial performance index (RVMPI) 0.27 (range: 0.16–0.52) versus 0.23 (0.14–0.42, *P* = 0.03), 2) RV diameter 1.97 (1.6–2.5) versus 1.88 (1.6–2.2) cm (*P* = 0.001), and 3) left ventricular diameter 4.8 (3.9–5.7) versus 4.71 (4–5.4) cm (*P* = 0.04).

### Intravenous fluids.

138/143 (96.5%) patients, received IV fluids during their hospitalization. The most frequently prescribed fluid was Ringer's lactate (422/584, 72.3%), followed by normal saline (77, 13.2%) and gelofusine/colloid (54, 9.25%). Total fluid volumes ranged from 0.5 to 13.5 L, median 3 L (IQR: 0.5–8.5 L) and were given for a median of 3 days (IQR: 1–8, 1–9 days), starting on D5 (IQR: 2–9) and finishing on D8 (IQR: 4–15). On ≥ D10, 3/134 (2.2%) started IV fluids and 24/134 (17.9%) were still on IV fluids. Platelets were administered to 47 (32.9%) patients whose median nadir platelet count was 17,500/μL (range 2,000–50,000/μL). No blood transfusions were given.

## Discussion

In this prospective dengue study in adults from metropolitan Hanoi, almost all patients had non-severe dengue, many had received preadmission IV fluids, most had dengue warning signs, and four (~3%) developed severe dengue, manifest as shock, and AST values exceeding 1,000 IU/L.

Our low rate of decompensated shock is consistent with adult data from Trung and others.^13^ Shock was not seen in 111 patients with “intermediate” severity dengue (64 had transient narrow pulse pressures) who were managed in an intensive care unit and in 333 patients with “mild” dengue who were treated in their adult infection ward.^13^ Thus, shock is preventable with good fluid management whereas dengue complications such as severe hepatitis or encephalitis are beyond the control of clinicians.

About 60% of our patients had received IV fluids prior to admission and almost all received IV fluids during admission. The majority finished IV fluids by the end of the critical period but a small proportion started IV fluids late in their illness and almost 20% continued on IV fluids to D10 and beyond. On admission, ~20% of patients had soft tissue edema and ~3% ascites/pleural effusions (A/PE), rising during admission to 38% and 23%, respectively.

The echocardiographic findings of impaired RV function and biventricular enlargement were consistent with fluid overload, and contrast the findings of normal RV function and smaller ventricular dimensions on admission versus discharge reported by Yacoub and others from southern Vietnam.^6^ Overhydration may explain partly the low rates of shock and low pulse pressures, but would have enhanced the clinical ill effects of plasma leakage. Pathophysiological studies are needed to advance our understanding of the complex interplay of fluid dynamics and cardiovascular instability in dengue, and give guidance on fluid management beyond the general recommendation^30^ of using IV fluids judiciously.

Adopting a proactive policy of IV fluids almost certainly resulted in some of our patients receiving IV fluids unnecessarily. Trung and others gave IV fluids to only 30% patients with mild dengue in the infection ward. Identifying non-severe dengue patients who could be managed adequately with oral fluids requires randomized trials of IV versus oral fluids. Such data would be helpful for clinics and general wards where detailed patient monitoring is too challenging.

Mucosal bleeding occurred in 45% of our patients, similar to the rate reported by Trung and others in their series.^13^ It was independent of the measured clotting factors, according with findings in non-severe dengue in Vietnamese children.^14^ Platelet dynamics were similar in those with and without mucosal bleeding and about one-third of patients received platelet transfusions. This is high compared with other studies that had good clinical outcomes with only very occasional platelet transfusion to patients with shock or severe bleeding.^13^ Platelets are relatively expensive and are being used without clear evidence of benefit. A randomized placebo-controlled trial is required.^31^

Our study took place during the dengue season when the prior probability of dengue was high. The antibody responses were essentially the same between NS1 antigen—PCR positive and negative groups. In the latter, we followed the 2009 WHO guidelines but also included patients with declining IgM titers when the convalescent sample was taken after D14, the mean time to peak IgM titers. If validated by others, this criterion could be incorporated into future WHO dengue guidelines.

Our study had limitations. It was conducted in an adult referral hospital, and the relatively small number of patients came mostly from Hanoi, a city with good medical facilities. These biases limit the applicability of our results. Ultrasound data were not collected systematically to monitor the changes in plasma leakage, and the severity of mucosal bleeding was not classified.

To conclude, non-severe dengue dominated the clinical picture in our setting and a significant proportion of patients developed evidence of fluid overload. More research is needed in community and hospital settings to optimize the fluid management, broaden our knowledge of dengue epidemiology, and assess their epidemic preparedness.

## Supplementary Material

Supplemental Table.

## Figures and Tables

**Table 1:** History, symptoms, and signs at enrollment in 143 patients with confirmed dengue*†

Parameter	Data
Demographic data	
Sex M:F	77:66
Age	23.5 (17–58, 16–72)
Hanoi resident	127/137 (92.7%)
Past medical history	
Previous dengue	6/141 (4.3%)
JE vaccine	30/135 (22.2%)
Previous JE illness	0/141
Symptoms	
Fever	142/142 (100%)
Headache	131/141 (92.9%)
Myalgia	106/138 (76.8%)
Rash	71/137 (51.8%)
Vomiting	64/139 (46.1%)
Cough	62/138 (44.9%)
Eye pain	54/135 (40.0%)
Bleeding	50/138 (36.2%)
Diarrhea	48/139 (34.5%)
Itch/prickly skin	38/134 (28.4%)
Abdominal pain	39/139 (28.1%)
Shortness of breath	9/135 (6.7%)
Physical signs	
Axillary temperature (°C)	37.8 (36–40, 36–40.2)
Pulse (b/min)	86 (64–125, 60–144)
Systolic BP mmHg	102.5 (90–130, 85–130)
Diastolic BP mmHg	70 (50–80, 50–80)
Abdominal tenderness	10/90 (10.9%)
Lethargy	3/143 (2.1%)
Facial/hand/ankle swelling	31/143 (21.7%)
Mucosal bleeding	28/143 (19.6%)
Palpable liver	5/143 (3.5%)
Laboratory results	
Hct (%)	42.5 (33.1–50.9, 30.3–52.8)
Platelet count	60 (12–126, 6–220)

Hct = hematocrit; JE = Japanese encephalitis.

* Proportional data are shown as numerators/denominators.

† Continuous data are shown as median, interquartile range, and full range.

**Table 2:** Vital signs and laboratory parameters recorded by illness days*†

Illness day	1	2	3	4	5	6	7	8	9	10	11	12	13	≥ 14
Patient numbers	4	16	47	87	114	132	127	120	86	45	28	11	6	75
Signs														
Temperature (°C)	38 (37.2–40)	38.5, 37.8–39.4 (36.6–40.6)	38, 36.8–39.5 (36–40)	38, 36.5–40 (36–40.2)	37.1, 36–40 (36–40)	36.8, 36–39 (36–39)	36.6, 36–38.6 (36–39)	36.5, 36–37.4 (36–38)	36.5, 36–37.2 (35.8–36.8)	36.5, 36–37.2 (36–38.5)	36.5, 36–36.8 (36–37.5)	36.5, 36.3–36.6 (36–37.8)	35.8, 36.6, 37.8	36.5, 36–37 (36–37.8)
Pulse rate/minute	95 (80–144)	100 (74–112)	88 (58–112)	88 (62–128)	84 (60–128)	80 (52–120)	80 (52–106)	78 (58–94)	78 (60–95)	78 (60–105)	80 (55–90)	80 (76–85)	76, 76, 82	80 (37–92)
Systolic BP (mmHg)	110 (90–120)	110 (90–110)	110 (90–130)	100 (85–128)	100 (85–120)	100 (80–130)	100 (80–130)	100 (90–120)	100 (85–140)	100 (70–130)	105 (90–140)	110 (100–120)	105 (100–110)	110 (90–140)
Diastolic BP (mmHg)	70 (50–80)	70 (60–80)	70 (60–80)	70 (50–90)	60 (50–90)	65 (50–80)	60 (50–90)	60 (50–80)	70 (50–90)	70 (50–80)	70 (70–80)	70 (60–70)	65 (60–70)	70 (60–90)
Pulse pressure (mmHg)	40 (30–40)	40 (30–40)	40 (25–60)	40 (25–65)	40 (20–55)	40 (20–60)	40 (20–70)	40 (30–60)	40 (25–60)	40 (20–60)	40 (30–60)	40 (30–50)	40 (30–50)	40 (30–50)
Hematological parameters														
Total white cell count × 103/μL	1.35, 3.5, 4.14, 8.84	2.57, 20.4–4.03 (1.27–4.36)	2.45, 1.7–4.92 (1.2–5.8)	2.55, 1.1–6.54 (0.89–8.51)	2.09, 1.43–6.85 (1.16–8.34)	4.56, 2.1–8.48 (1.57–10.55)	5.05, 2.5–10.9 (2.02–12.4)	5.43, 3.09–11.62 (2.53–15.9)	5.31, 3.29–8.28 (2.61–9.51)	4.93, 3.84–7.49 (3.64–8.84)	6.26, 4.83–6.5 (3.79–9.73)	5.21, 5.45, 7.11, 8.33	3.83, 5.49, 7.27	6.75, 4.01–9.2 (3.36–18.3)
Absolute neutrophil count/μL	677; 940; 2,724; 7,478	1,170; 1,002–2,500 (239–3,163)	1,299; 591–2,799 (400–4,261)	1,080; 479–3,150 (330–4,032)	1,050; 260–2,829 (178–3,679)	1,038; 432–3,112 (330–4,552)	1,326; 319–4,595 (250–6,890)	1,476; 511–3,643 (218–10,176)	1,728; 682–3,486 (312–4,363)	1,699; 1,124–3,462 (647–4,031)	1,994; 1,624–3,016 (1,336–3,876)	1,705; 2,104; 2,787; 3,540	1,731; 2,300; 3,576	3,395; 1,982–4,950 (67–15,207)
Absolute lymphocyte count/μL	179; 663; 919; 2,520	615; 568–1,018 (360–1,790)	775; 490–1,989 (410–2,575)	938; 349–2,642 (250–5,157)	1,390; 530–3,897 (360–5,203)	2,299; 739–4,754 (480–6,086)	2,531; 1,111–6,108 (998–6,210)	2,485; 1,320–5,291 (899–5,332)	2,401; 1,371–4,419 (910–6,210)	2,046; 1,488–3,167 (858–3,702)	2,115; 1,729–2,355 (1,550–3,618)	1,781; 2,630; 2,752; 3,073	1,639; 2,141; 2,188	2,288; 1,317–3,569 (40–4,433)
Neutropenia n/d (%)	2/3 (66.6)	7/12 (58.3)	30/43 (69.8)	55/81 (67.9)	82/108 (75.9)	86/127 (67.7)	73/119 (61.3)	54/112 (48.2)	32/79 (40.5)	10 (29.4)	2/13 (15.4)	0	0	1/57 (1.75)
Lymhopenia n/d (%)	2/3 (66.6)	8/12 (75)	30/43 (69.7)	46/81 (56.8)	30/108 (27.8)	9/127 (7.1)	1/119 (0.8)	1/112 (0.9)	1 /79 (1.3)	1/34 (2.9)	0	0	0	2/57 (3.5)
Atypical lymphocytes n/d (%)	0	1/12 (8.3)	6/43 (13.95)	18/81 (22.2)	12/108 (11.1)	6/127 (4.7)	6/119 (5.04)	20/112 (17.8)	34/79 (43.04)	18/34 (52.9)	13 (23.08)	4/4 (100)	0	1/57 (1.75)
Immature neutrophils n/d (%)	0	0	2/43 (4.6)	8/81 (9.9)	19/108 (17.6)	19/127 (14.9)	13/119 (10.9)	12/112 (10.7)	16/79 (20.2)	7/34 (20.6)	3/13 (23.1)	2/4 (50)	0	2/57 (3.5)
Biochemical parameters														
AST (IU/L)	37, 39, 87, 479	60, 64–75 (24–255)	115, 50–250 (29–599)	131, 60–455 (21–1,120)	113, 44–773 (6–1,055)	125, 73–338 (52–721)	164, 122–282 (25–1,126)	140, 72–372 (60–437)	191, 72–346 (50–485)	134, 123–265 (57–486)	126.5, 83–153 (3.6–407)	68, 88, 118, 166, 320	30, 121	50, 29–141 (27–230)
ALT (IU/L)	16, 25, 64, 328	61, 27–73 (18–132)	72, 32–180 (12–306)	69, 19–264 (10–683)	67, 22–360 (14–786)	73, 45–236 (30–734)	105, 54–222 (14–626)	138, 75–401 (45–506)	231, 63–434 (27–502)	251, 103–374 (73–607)	221, 149–268 (39–432)	55, 174, 244, 350, 351	64, 288	84, 35–278 (21–418)
Total bilirubin	6	16.4	8.55, 4.7–12 (3.4–21.1)	7.1, 5.4–13 (3.9–28.6)	7.35, 4.5–13 (3.9–14)	9.9, 5–16.3 (3.2–18)	10, 6.5–16.5 (5–21.7)	8, 5–15 (3.6–17)	10.3, 6.5–15.5 (4.3–22.4)	10.9, 7–12 (3.9–14.1)	8.5, 8–9.5 (5.9–10.9)	11.8, 15	5.8, 16.9	10.5, 6.7–29.3 (5.1–88)
Albumin (g/L)	42	ND	48, 45–51 (35–55)	47, 44–51 (35–59)	45, 40–52 (36–55)	47, 42–53 (36–56)	45, 43–52 (38–55)	49, 44–52 (41–57)	50.5, 45–53 (42–57)	49, 48–51 (42–56)	48, 47–50 (42–53)	48, 51	51	54, 49–59 (48–63)
Blood clotting parameters														
aPTT (seconds)	32.9	26.7, 35.3	33.3, 31.2–38.8 (29.5–43.2)	34.8, 31.5–39.5 (29.1–50.3)	36.5, 29.4–45 (25.2–65.1)	32.2, 28.4–41.5 (23.5–45)	30.1, 27.9–33.2 (24–38.6)	27.6, 23.8–32.4 (20.7–34)	27.7, 24.4–28 (22.8–30.4)	25.9, 25.3–26.8 (21.6–28.9)	25.9, 24.8–26.6 (23.5–30.5)	23.9	26.8	25.9, 28.3, 28.4
PT (%)	116.2	78.2, 114.4	105.9, 91.7–128.1 (81.3–155.5)	114.4, 98.4–126 (82.4–134.8)	120.9, 97–142 (79.2–158.4)	126, 97–160 (90.5–167.7)	134.8, 118.1–147.2 (101.3–152.6)	130.3, 109.2–149.9 (95.7–171.6)	132.4, 107.6–155 (73.6–161)	123.9, 112.6–126 (91.7–152.6)	129.2, 116.2–132.5 (88–144.1)	121.9	149.9	116.4, 142, 152.6
Fibrinogen	1.5	0.9, 2.5	1.76, 1.24–2.07 (0.9–2.54)	1.4, 1.2–2.1 (0.96–3.13)	1.55, 1–2.17 (0.9–2.4)	1.6, 1.23–2.47 (0.39–2.6)	1.6, 1.2–1.97 (0.33–2.5)	1.75, 1.4–2.53 (1.16–19.6)	1.65, 1.5–2.19 (1.14–2.99)	1.85, 1.7–2.2 (1.33–3.7)	1.5, 1.3–2 (1.12–2.5)	1.2	1.4	1.4, 1.6, 1.95

ALT = alanine transaminase; aPTT = activated partial thromboplastin time; AST = aspartate aminotransferase; BP = blood pressure; ND = no data; PT = prothrombin time.

* Pulse rate and BP data are shown as median (full range); all other continuous data as median, interquartile range, and (full range).

† ≥ D14 includes data on and after illness day 14.
