# Supplementary material for: Dengue in Adults Admitted to a Referral Hospital in Hanoi, Vietnam
Source: Am J Trop Med Hyg. 2015 Jun 3;92(6):1141–9. doi: 10.4269/ajtmh.14-0472 (PMC4458817; doi:10.4269/ajtmh.14-0472)
Supplement: Supplementary file 1 [file SD2.pdf]

Supplemental Table 1

Clinical features of five patients who had pulse pressures of 20 mmHg

Age	Sex	Illness day	Temperature (°C)	Pulse rate b/min	Cool hands or feet	Swelling	Mucosal bleeding	Abdominal tenderness	Abdominal pain	Enlarged liver	Plasma leakage	Hematocrit (%)*
19	M	5	36.5	84	N	N	N	N	Y (D6)	N	N	43.3
25	M	6	36.4	72	N	N	ND	N	N	N	N	45.3
30	M	6	38.3	72	Y	N	ND	N	Y (D7)	N	N	47.7
72	F	7	36	88	N	N	Vagina (D4–D8)	N	N	N	N	41.5
23	F	10	36.2	ND†	N	Face (D6)	ND	N	Y (D7)	ND	Ascites (D6)	42.2

F = female; M = male; N = no; ND = no data; Y = yes.

*Hematocrit on the day the pulse pressure was 20 mmHg.

†On D9: pulse = 76/min, blood pressure (BP) = 85/60.
